# Integrated Transcriptome and Methylome Analyses Reveal Sex-Specific Molecular Responses to Chronic Heat Stress in Tongue Sole (*Cynoglossus semilaevis*)

**DOI:** 10.3390/ani16132078

**Published:** 2026-07-05

**Authors:** Yangzhen Li, Wenteng Xu, Xinqi Wen, Ailing Wu, Hongxiang Zhang, Haien Zhang, Weidong Li, Songlin Chen

**Affiliations:** 1 State Key Laboratory of Mariculture Biobreeding and Sustainable Goods, Yellow Sea Fisheries Research Institute, Chinese Academy of Fishery Sciences, Qingdao 266071, China; liyz@ysfri.ac.cn (Y.L.); xuwt@ysfri.ac.cn (W.X.);; 2 Laboratory for Marine Fisheries Science and Food Production Processes, Qingdao Marine Science and Technology Center, Qingdao 266237, China; 3 Tangshan Haidu Seafood Co., Ltd., Tangshan 063506, China; 4 National Demonstration Center for Experimental Fisheries Science Education, Shanghai Ocean University, Shanghai 201306, China; 5 Fishery Research Institute, Tangshan Academy of Agricultural Sciences, Tangshan 063001, China

**Keywords:** chronic heat stress, tongue sole, DNA methylation, RNA-seq, sex-specific response

## Abstract

Chronic heat stress poses a major challenge to marine aquaculture, yet its sex-associated molecular basis remains poorly understood in Chinese tongue sole (*Cynoglossus semilaevis*). Juveniles were exposed to a control temperature (24 °C) or elevated temperature (30 °C) for two months, and liver samples from low-temperature females (LF), high-temperature females (HF), low-temperature males (LM), and high-temperature males (HM) were analyzed by RNA sequencing (RNA-seq) and whole-genome bisulfite sequencing (WGBS). Principal component analysis indicated a strong temperature-associated separation, while sex-related separation under heat stress was smaller and was mainly observed along the second principal component. In high-temperature relative to low-temperature comparisons, females showed a broader set of differentially expressed genes (DEGs; LF vs. HF, 1968) than males (LM vs. HM, 506). Differential methylation analyses indicated that cytosine-guanine (CG) methylation was the predominant heat-associated epigenetic signal. Integrative analysis identified 624 overlapping genes between DEGs and CG-associated differentially methylated genes (CG-DMGs) in females and 177 in males, suggesting broader methylation-associated transcriptional remodeling in females. Functional enrichment associated the female overlap genes with immune response, inflammatory signaling, lipid metabolism, and detoxification, whereas male overlap genes were more closely associated with proteostasis, autophagy, and DNA replication/repair. Correlation analyses suggested modest methylation–expression coupling and highlighted candidate W-linked genes, including H2AZ2 and ANKRD13A. Overall, these results should be regarded as a preliminary baseline for understanding sex-associated molecular responses to chronic heat stress in tongue sole and as a source of candidate genes and pathways for future validation and heat-resilience breeding.

## Simple Summary

Chronic high temperature is becoming an increasing challenge for marine aquaculture, but the biological responses of male and female fish may not be the same. In this study, juvenile tongue sole were kept at normal or elevated water temperature for two months, and liver samples from males and females were analyzed to understand how long-term heat exposure affects gene activity and chemical regulation of the genome. The results showed that females had a much broader response to heat stress than males. In females, heat stress mainly affected immune response, inflammation, lipid metabolism, and detoxification, whereas in males, the response was more related to protein maintenance, cellular recycling, and DNA repair. Several genes located on the female-specific chromosome were also identified as potential contributors to female-biased heat responses. These findings improve our understanding of how tongue sole cope with long-term heat stress and provide useful candidate genes and pathways for breeding heat-resilient aquaculture strains.

## 1. Introduction

Global climate change has led to an increase in the frequency and intensity of extreme weather events, including marine heatwaves, which pose significant threats to aquatic ecosystems and aquaculture industries worldwide [1,2,3,4,5,6]. Aquatic ectotherms, such as fish, are particularly vulnerable to elevated temperatures due to their reliance on external thermal conditions to regulate physiological processes [7,8,9,10,11]. Exposure to chronic or acute heat stress can result in adverse effects on growth, reproduction, immune function, and survival, thereby impacting both wild populations and aquaculture productivity [12,13,14,15,16,17].

In aquaculture, enhancing the heat tolerance trait in cultured species has become a critical objective to mitigate the impacts of rising water temperatures. While considerable research has focused on cold- and cool-water species, warm-water species are not exempt from heat-induced challenges [18]. High temperatures can negatively affect growth rates, disease resistance, and even induce sex reversal in certain fish species [19,20,21], implying the importance of understanding heat stress responses across diverse thermal niches. It has been extensively reported that marine heatwaves resulted in mass mortality events in various aquaculture species, including finfish and bivalves [10,22,23,24,25]. These events underscore the urgent need for strategies to enhance the resilience to thermal stress in aquaculture.

Epigenetic mechanisms, particularly DNA methylation, have emerged as important regulators of gene expression in response to environmental stimuli, including temperature fluctuations, as demonstrated in several fish species [26,27,28,29]. DNA methylation can modulate gene activity without altering the underlying genetic code, thereby contributing to phenotypic plasticity and potentially helping organisms acclimate to changing environments [30,31,32]. In fish, recent studies have shown that even modest temperature increases can be associated with transient or persistent shifts in DNA methylation patterns, which may be linked to changes in the expression of genes involved in stress responses, metabolism, and development [33,34,35].

Tongue sole (*Cynoglossus semilaevis*) exhibits pronounced sexual dimorphism, with adult females growing significantly larger and faster than males, where mature females can reach body weights up to six times greater than their male counterparts [36,37,38,39]. Another distinctive biological feature of this species is its sensitivity to temperature, particularly during the thermosensitive period of early development (from 30 to 100 days post-hatch) [40]. Exposure to elevated temperatures during this stage (e.g., 28 °C) can induce sex reversal, causing genetic females to develop as phenotypic males, known as pseudomales, which is primarily regulated by DNA methylation [41]. These characteristics make tongue sole an ideal model organism for studying vertebrate sex determination and differentiation.

Beyond that, tongue sole is a commercially important flatfish species widely farmed in China [42,43]. As an eurythermal fish, tongue sole is valued for its adaptability to a broad range of water temperatures (from 5 to 30 °C) and for the rapid growth of females, which are preferentially cultured in aquaculture systems under warm waters with optimum temperatures ranging from 22 to 24 °C. Despite this, the molecular mechanisms underlying its response to chronic heat stress remain poorly characterized. Given the increasing threat of marine heatwaves due to climate change, understanding the molecular basis of thermal adaptation in tongue sole is of both scientific and practical importance.

In this study, we aimed to determine whether chronically elevated temperature induces sex-associated transcriptomic and DNA methylation responses in tongue sole liver and to identify candidate genes and pathways linked to thermal adaptation. We hypothesized that chronic heat exposure would alter both gene expression and DNA methylation and that these responses would differ between females and males because of the pronounced sexual dimorphism and temperature-sensitive sex-related biology of this species. By integrating RNA-seq and WGBS data, we sought to identify methylation-associated transcriptional changes that may provide candidate markers for future validation and heat-resilience breeding.

## 2. Materials and Methods

### 2.1. Heat Stress and Sample Collection

Juvenile tongue sole (10–12 cm in total length) were randomly selected from the 2022 year-class family-based selective breeding nucleus population at Haidu Seafood Co., Ltd. (Tangshan City, China). The 3000 fish used for the temperature-rearing experiment were part of a larger ongoing commercial breeding evaluation rather than a population established solely for this molecular study. Fish were distributed evenly into two 6 × 6 × 0.8 m tanks (1500 fish per tank). After a 2-week acclimation, the water temperature of the control group was maintained at 24 ± 0.5 °C, which is close to the normal culture temperature for tongue sole, whereas the treatment group was gradually elevated to 30 ± 0.5 °C at 1 °C per day to impose chronic high-temperature stress near the upper range used in culture. The water exchange rate was 100% per day, and feeding, aeration, and other husbandry conditions were kept identical between groups. After two months, 10 individuals from each group were randomly sampled for tissue collection. Liver samples were used for transcriptome and methylome analyses, fin clips were used for DNA-based genetic sex identification, and gonads were inspected to confirm phenotypic sex and exclude pseudomales. Samples were flash-frozen in liquid nitrogen and stored at −80 °C until use. Before sampling, fish were anesthetized with MS-222 (tricaine methanesulfonate; 10 mg/L) to minimize suffering.

Fin clips were used for DNA extraction and genetic sex identification using markers and methods developed in our previous study [44]. To examine and exclude pseudo-males, phenotypic sex was further confirmed by optical inspection of the gonads. Finally, liver samples from two males and two females in each temperature group were selected for matched RNA-seq and WGBS analyses. The sample names of male (M) and female (F) individuals in low-temperature (L) and high-temperature (H) groups were labeled as LM1, LM2, LF1, LF2, HM1, HM2, HF1, and HF2, respectively. Although the original design intended to compare temperature groups with pooled sexes (*n* = 4 per group), sex-stratified analyses were subsequently adopted after preliminary PCA suggested sex-associated clustering under heat stress. Because each sex-temperature subgroup contained two biological replicates, the multi-omics results should be interpreted as a preliminary baseline and candidate-generating dataset rather than as conclusive population-level evidence.

### 2.2. RNA Sequencing and Transcriptomic Analysis

Total RNA was extracted from liver samples using TRIzol Reagent commercial kit (Invitrogen, Carlsbad, CA, USA). After quality assessment, quantification, and fragmentation, cDNA libraries were prepared using the Illumina RNA-seq Library Prep Kit and sequenced on the Illumina HiSeq X Ten System to generate paired-end 150 bp reads. The pipeline for identifying differentially expressed genes (DEGs) was similar to our previous studies [45]. In brief, raw reads were subjected to quality control using fastp (v1.0.0) to remove adapters and low-quality reads, generating clean reads [46]. Clean reads were then aligned to the tongue sole reference genome using HISAT2 (v2.1.1) [47]. Gene-level raw counts were obtained using featureCounts (v2.1.1) [48] and used as input for the DESeq2 R package (v1.50.2) to identify DEGs [49]. Genes with |log2FoldChange| >= 1 and an adjusted false discovery rate (FDR) < 0.05 were considered significantly differentially expressed. Because the number of biological replicates was limited, DEG results were interpreted together with PCA, pathway enrichment, and methylation evidence as exploratory molecular patterns requiring further validation. For expression quantification and visualization, gene expression levels were additionally normalized as fragments per kilobase of transcript per million mapped reads (FPKM) using the RSEM algorithm [50].

### 2.3. Whole-Genome Bisulfite Sequencing

Genomic DNA was individually isolated from liver samples using DNeasy Blood & Tissue Kits (QIAGEN, Valencia, CA, USA) following the manufacturer’s instructions. DNA concentration and integrity were assessed using a NanoDrop spectrophotometer and Agarose Gel Electrophoresis, respectively. Extracted DNA (1 µg) was spiked with 1 ng of fragmented unmethylated phage λ DNA (Promega, Madison, WI, USA, average size: 300 bp). Then, the DNA was fragmented to an average size of 150–300 bp using a Covaris sonicator and purified using the MiniElute PCR Purification Kit (QIAGEN, Germantown, MD, USA). Fragmented DNA was end-repaired, phosphorylated, and A-tailed, and methylated sequencing adapters were ligated using the TruSeq DNA Sample Prep Kit (Illumina, San Diego, CA, USA). Bisulfite conversion was then performed using the EpiArt^®^ DNA Enzymatic Methylation Kit (Vazyme Biotech, Nanjing, China), following the manufacturer’s protocol. The converted DNA fragments were PCR amplified and purified with AMPure XP beads (Beckman Coulter, Brea, CA, USA). Library quality and size distribution were confirmed using an Agilent 2100 Bioanalyzer (Agilent Technologies, Santa Clara, CA, USA). The final libraries were quantified (concentration > 2 nM) and sequenced on an Illumina NovaSeq X Plus platform.

### 2.4. Methylation Analysis

Raw sequencing reads were filtered using Trimmomatic software [51] with default parameters. The resulting clean reads were then mapped to the tongue sole reference genome using BSMAP (v2.90) [52] with default settings. Methylated cytosines were identified using a custom Perl script, followed by statistical correction using the algorithm described by Lister et al. [53]. The methylation level was calculated as the percentage of methylated cytosines across the whole genome, within each chromosome, and in different genomic regions for each sequence context (CG, CHG, and CHH). To explore methylation patterns in various genomic regions, we profiled the methylation levels in 2 kb flanking regions and gene bodies, presenting the average methylation level for each window.

Differential DNA methylation between groups was analyzed with methylKit [54], using a minimum read coverage of 4 to determine the methylation status of individual cytosines. Significant differences in methylation levels were identified using Pearson’s chi-square test (χ^2^), with a false discovery rate (q-value) threshold of ≤0.05. Criteria for differentially methylated cytosines (DMCs) are as follows: for CG and CHG contexts, |Δmethylation ratio| ≥ 0.20; for CHH context, |Δmethylation ratio| ≥ 0.10; for all contexts combined, |Δmethylation ratio| ≥ 0.15. Sliding windows of 200 bp were used with the following context-specific thresholds to identify differentially methylated regions (DMRs). The criteria are as follows: for CG and CHG contexts, windows containing ≥ 5 CGs or CHGs, |Δmethylation ratio| ≥ 0.20; for CHH context, windows containing ≥ 15 CHHs, |Δmethylation ratio| ≥ 0.10; for all contexts combined, windows containing ≥ 20 cytosines, with |Δmethylation ratio| ≥ 0.15. Genes containing DMRs or located within 2 kb of DMR boundaries were classified as differentially methylated genes (DMGs). Gene annotations from the tongue sole genome were integrated into the analysis using bedtools (V2.31.1) [55].

### 2.5. Integrative Analysis of RNA-Seq and WGBS

To explore potential methylation–expression associations under chronic heat stress, an integrative analysis was conducted by combining RNA-seq and WGBS data from liver samples of tongue sole. DEGs identified from RNA-seq were cross-referenced with DMGs derived from WGBS to identify overlapping genes associated with both transcriptional and DNA methylation changes. For each overlapping gene, the average methylation changes (Δmethylation ratio) within defined genomic regions (upstream 2 kb, gene body, and downstream 2 kb) were calculated, and Pearson’s correlation analysis was performed between gene expression levels and methylation levels. Although methylation differences were initially evaluated across cytosine contexts, integrative overlap, enrichment, and correlation analyses were performed primarily using CG-DMGs because CG methylation represented the dominant and most interpretable signal. These analyses were used to identify methylation-associated candidate genes and do not by themselves demonstrate direct methylation-mediated regulation.

### 2.6. Enrichment Analysis

To investigate the biological significance of transcriptional and epigenetic changes in response to chronic heat stress in tongue sole, DEGs and CG-DMGs were subjected to Gene Ontology (GO) and Kyoto Encyclopedia of Genes and Genomes (KEGG) pathway enrichment analyses. Enrichment was assessed using the hypergeometric test, GO terms and KEGG pathways with a q-value < 0.05 were considered significantly enriched.

### 2.7. Visualization of RNA-Seq and WGBS Results

Principal Component Analysis (PCA) was performed on RNA-seq expression data and methylation data (CG context, coverage >10) to evaluate the variance across samples and their clustering patterns using the ggplot2 (v3.5.1) R package. Bar plots were generated using the clusterProfiler (v4.4.4) R package to show the top enriched GO terms and KEGG pathways based on q-values. Bubble plots were created using ggplot2 (v3.5.1) R package to visualize the relationships among enriched terms, with bubble size representing the number of genes and color indicating statistical significance. Scatter plots were generated using the ggplot2 (v3.5.1) R package to visualize the relationship between regional CG methylation changes and gene expression changes of overlapping genes. In each plot, each point represented one gene, with the *x*-axis indicating CG methylation difference (Δmethylation) and the *y*-axis indicating transcriptomic change [log2(fold change)]. For pairwise labels used, positive expression or methylation differences indicate higher values in the second group relative to the first group (for example, HF relative to LF for LF vs. HF, and HM relative to LM for LM vs. HM). Fitted linear regression lines were added to illustrate the overall correlation trend.

## 3. Results

### 3.1. Overview of RNA-Seq and WGBS Data

For RNA-seq, liver transcriptomes from eight individuals (2 females and 2 males per temperature group) were sequenced, yielding 42–70 (average 63.51) million clean reads per sample, with average Q20 and Q30 scores of 97.71% and 93.44%, respectively (Table S1). The average mapping rate to the reference genome was 94.02%, and 88.89% of mapped reads aligned to exonic regions, confirming effective mRNA enrichment. For WGBS, an average of 109.28 million clean reads was obtained per sample after quality control, corresponding to an average genome coverage of 24.6× and a mapping rate of 80.98% (Table S2). Bisulfite conversion rates, estimated using spiked-in lambda phage DNA, ranged from 98.59% to 98.99%, indicating high experimental fidelity and minimal non-conversion bias. Global DNA methylation patterns revealed the presence of three methylation contexts, CG, CHG, and CHH (where H = A, C, or T) in tongue sole liver tissue. Among these, CG methylation was predominant, with average genome-wide methylation levels around 70%, whereas CHG and CHH methylation levels were much lower, both below 10% across all samples (Table 1).

### 3.2. Principal Component Analysis Based on Gene Expression and DNA Methylation

**Figure 1:**
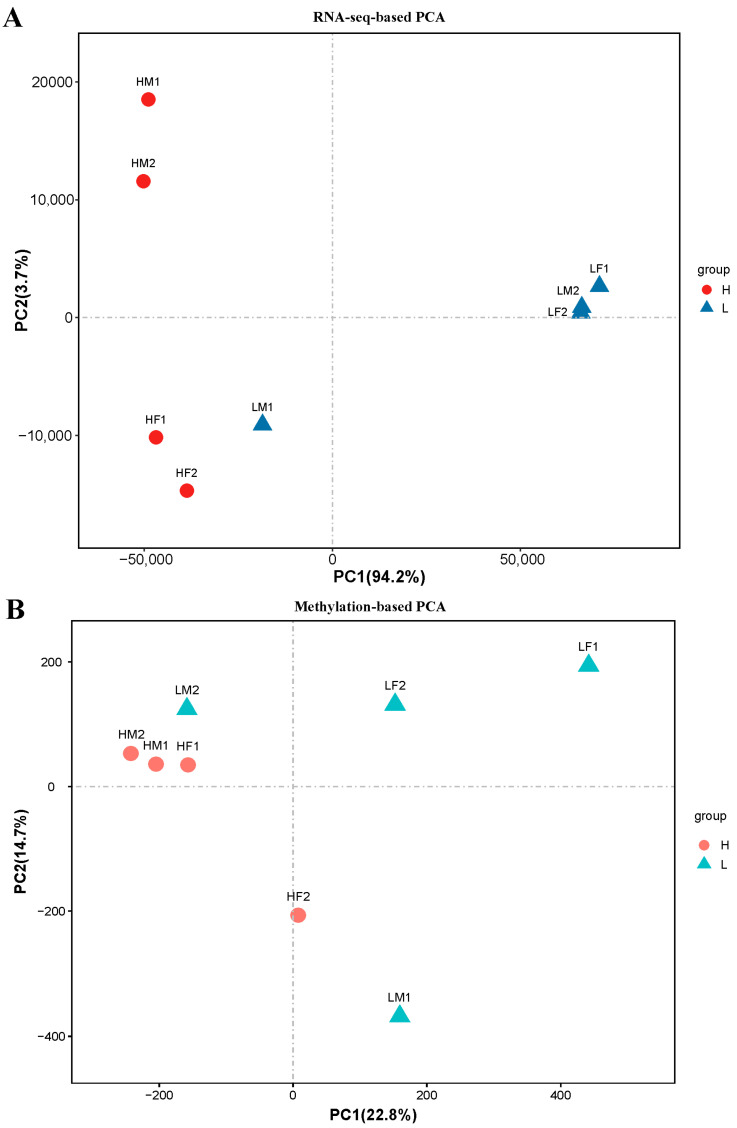
Principal component analysis (PCA) of transcriptomic and DNA methylation profiles in liver samples of tongue sole under low- and high-temperature conditions. (**A**) PCA based on RNA-seq gene expression profiles. (**B**) PCA based on CG methylation levels of sites. Each point represents one individual sample, including high-temperature females (HF1, HF2), high-temperature males (HM1, HM2), low-temperature females (LF1, LF2), and low-temperature males (LM1, LM2). Samples are colored according to temperature group, with H indicating high temperature and L indicating low temperature. The percentages shown on the axes represent the proportion of total variance explained by the corresponding principal components.

PCA based on liver transcriptomes (Figure 1A) and CpG methylation profiles (>=10× coverage; Figure 1B) revealed a strong temperature-associated separation among tongue sole liver samples. RNA-seq-based PCA showed that the first principal component (PC1) accounted for 94.2% of the total variance and separated samples primarily by temperature. Within the high-temperature group, females and males showed additional separation along PC2, which accounted for 3.7% of the total variance; therefore, this pattern was interpreted as a secondary sex-associated trend under heat stress rather than evidence of a large global sex effect. In contrast, low-temperature samples clustered closely together, indicating more uniform transcriptomic profiles under control conditions. Methylation-based PCA revealed a distinct pattern, where high-temperature samples (HF and HM) clustered more tightly, while low-temperature samples (LF and LM) were more dispersed, indicating greater inter-individual variability in DNA methylation under non-stress conditions. This reduction in epigenetic variability under chronic heat stress may reflect a coordinated epigenetic response to thermal challenges. Overall, these PCA results suggest that both transcriptional and epigenetic landscapes in the liver are strongly associated with temperature, with a smaller sex-associated divergence observed under heat stress.

### 3.3. Analysis of Differentially Expressed Genes

**Figure 2:**
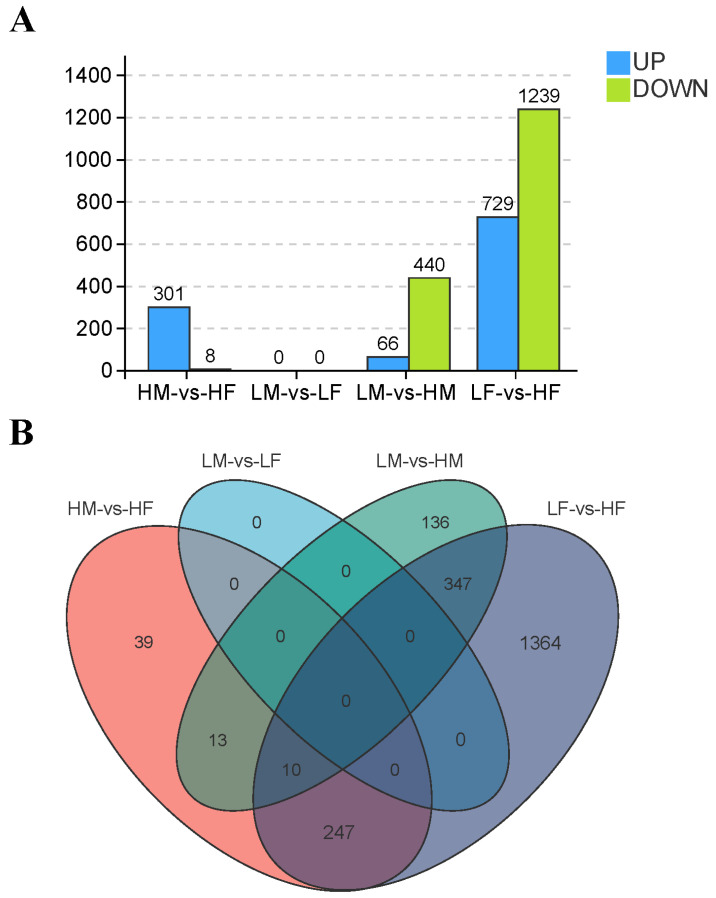
Differentially expressed genes (DEGs) across sex and temperature comparisons in tongue sole liver. (**A**) Numbers of upregulated (UP) and downregulated (DOWN) DEGs identified in the four pairwise comparisons: HM vs. HF, LM vs. LF, LM vs. HM, and LF vs. HF. (**B**) Venn diagram showing the overlap of DEGs among the four comparisons.

Based on the sex-associated trend observed in the PCA of gene expression profiles under high-temperature conditions, sex-stratified differential expression analysis was performed to explore transcriptional responses to chronic heat stress. Gene expression profiles were compared between high- and low-temperature groups within each sex. In females, 1968 DEGs were identified, including 729 upregulated and 1239 downregulated genes, while in males, 506 DEGs were detected, of which 66 were upregulated and 440 were downregulated (Figure 2A). These results suggest a broader transcriptional response in females than in males in this exploratory dataset. To further assess sex-based differences in gene expression under each temperature condition, direct comparisons were made between females and males. No DEGs were detected between LF and LM under control conditions, whereas 309 DEGs (301 upregulated and 8 downregulated) were identified between HF and HM under heat stress. These DEG patterns should be interpreted cautiously because of the limited number of biological replicates.

Venn diagram analysis further illustrated the context-specific nature of differential expression (Figure 2B). Overlaps among the four comparisons were limited, with the female heat contrast showing the largest DEG set and the low-temperature sex contrast showing little baseline divergence. These patterns suggest that transcriptional responses to chronic heat stress are influenced by both temperature and sex, although validation with larger replicated cohorts will be required.

**Figure 3:**
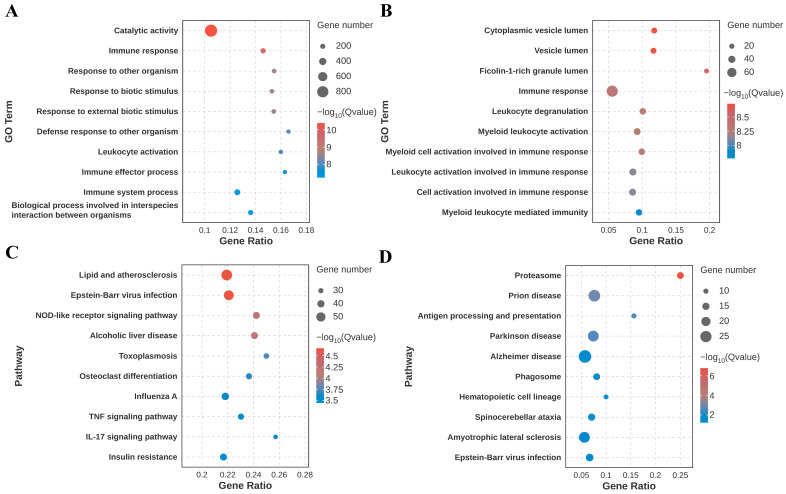
Bubble plots of Gene Ontology (GO) and Kyoto Encyclopedia of Genes and Genomes (KEGG) enrichment analyses of differentially expressed genes (DEGs) in female and male heat-response comparisons. (**A**) GO enrichment of DEGs in LF vs. HF. (**B**) GO enrichment of DEGs in LM vs. HM. (**C**) KEGG enrichment of DEGs in LF vs. HF. (**D**) KEGG enrichment of DEGs in LM vs. HM.

GO/KEGG enrichment of DEGs revealed strong sex- and temperature-dependent transcriptional responses in tongue sole liver. In females (LF vs. HF), DEGs were predominantly enriched for innate immune activation and inflammatory signaling (e.g., immune/defense response, leukocyte activation, cytokine regulation), together with stress and metabolic pathways including NOD-like receptor, TNF and IL-17 signaling, PPAR/insulin-related pathways, and cell fate programs (p53 signaling, apoptosis, and necroptosis), indicating coordinated immune–inflammatory remodeling coupled with metabolic reprogramming under heat stress (Figure 3A,C). In males (LM vs. HM), enrichment remained immune-centered but was more focused, highlighting leukocyte/neutrophil degranulation and vesicle/granule-associated functions, with KEGG signals dominated by proteasome, antigen processing and presentation, and phagosome, suggesting prioritization of proteostasis and immune surveillance (Figure 3B,D). Under heat-stress sex contrast (HM vs. HF), enriched terms shifted toward RNA processing (e.g., RNA splicing) and stress-response pathways (notably p53 signaling), supporting sex-biased regulation of transcriptional machinery under elevated temperature.

### 3.4. Differential Methylation Analysis

**Figure 4:**
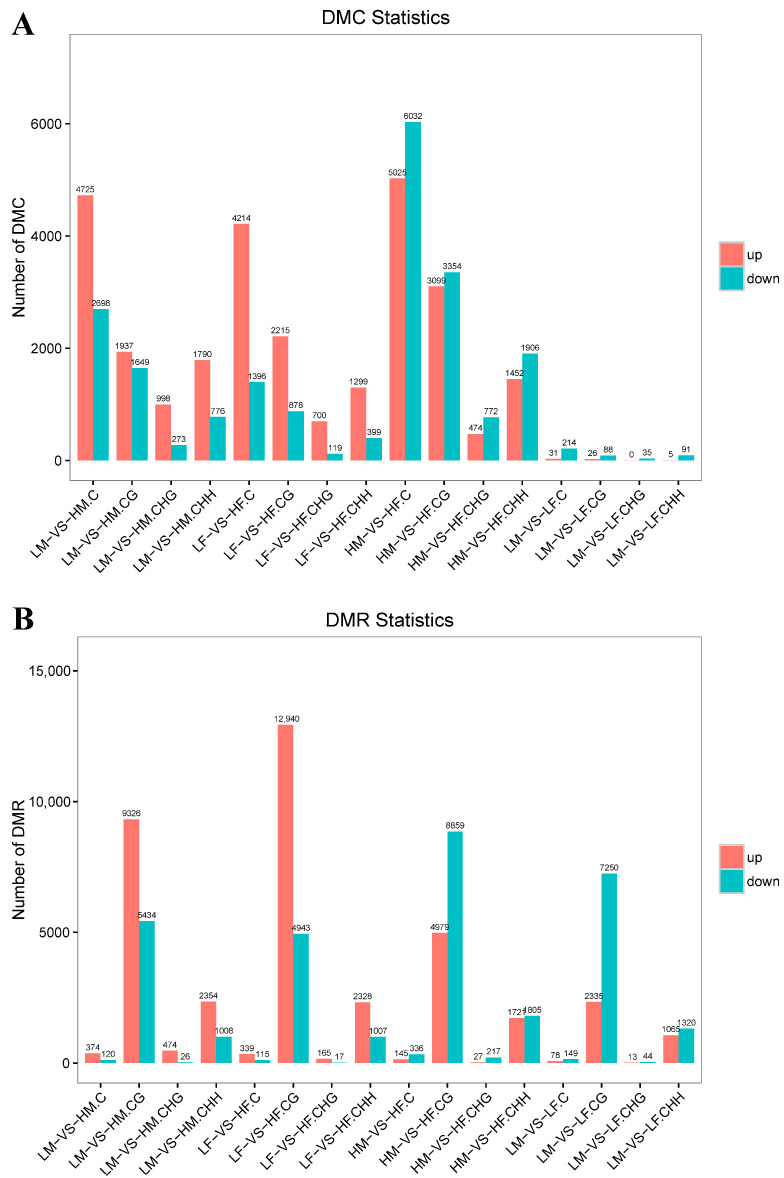
Numbers of differentially methylated cytosines (DMCs) and differentially methylated regions (DMRs) identified across four pairwise comparisons and four cytosine contexts in tongue sole liver. (**A**) Bar plot showing the numbers of up- and down-regulated DMCs in the C, CG, CHG, and CHH contexts for the comparisons LM vs. HM, LF vs. HF, HM vs. HF, and LM vs. LF. (**B**) Bar plot showing the numbers of up- and down-regulated DMRs in the same four methylation contexts and comparisons.

To characterize sex-associated methylation signatures associated with chronic heat stress in tongue sole liver, differential methylation was assessed across four pairwise comparisons (LM vs. HM, LF vs. HF, HM vs. HF, and LM vs. LF) and four cytosine contexts (C, CG, CHG, and CHH). In within-sex temperature contrasts, substantial methylation remodeling was observed. In males (LM vs. HM), 7423 DMCs were detected in the C context (4725 up; 2698 down), including 3586 CG-DMCs (1937 up; 1649 down). In females (LF vs. HF), 5610 C-context DMCs were identified (4214 up; 1396 down), including 3093 CG-DMCs (2215 up; 878 down) (Figure 4A). In both sexes, CG sites consistently accounted for the largest number of differential events compared with CHG and CHH, indicating that CG methylation is the major component of temperature-associated epigenetic variation in liver. Sex contrasts showed strong temperature dependence. Under heat stress (HM vs. HF), 11,057 C-context DMCs (5025 up; 6032 down) and 6453 CG-DMCs (3099 up; 3354 down) were detected, whereas under low temperature (LM vs. LF) only 245 C-context DMCs (31 up; 214 down) and 114 CG-DMCs (26 up; 88 down) were identified (Figure 4A), suggesting limited baseline sex differences but stronger sex-associated methylation divergence under heat.

Consistent with the DMC results, DMR patterns were dominated by the CG context. CG-DMRs were most abundant in the within-sex temperature contrasts (LM vs. HM: 9326 up/5434 down; LF vs. HF: 12,940 up/4943 down), while CHG-DMRs were rare and CHH-DMRs were intermediate (Figure 4B). In sex comparisons, CG-DMRs were skewed toward hypomethylation in both heat (HM vs. HF: 4979 up/8859 down) and low temperature (LM vs. LF: 2335 up/7250 down), indicating a distinct directionality relative to temperature contrasts. DMG annotation further supported the predominance of CG-associated changes (Table 2), with 4703–7356 CG-DMGs across comparisons, compared with 1422–1969 CHH-DMGs and only 38–57 CHG-DMGs.

GO and KEGG enrichment analyses of CG-DMGs across the four comparisons (LF vs. HF, LM vs. HM, HM vs. HF, and LM vs. LF) consistently pointed to methylation changes concentrated in membrane-associated signaling and cellular communication, including functions related to the cell periphery, cell adhesion, and signal transduction. In the within-sex heat contrasts (LF vs. HF and LM vs. HM), CG-DMGs were additionally enriched for immune-related processes (e.g., cytokine-mediated signaling, leukocyte activation/migration) and regulatory signaling terms (e.g., protein phosphorylation), together with KEGG pathways involved in core signal transduction (Rap1, phospholipase D, calcium, cGMP-PKG), and broader stress/metabolic regulation such as MAPK signaling, circadian entrainment, and endocrine modules (e.g., insulin secretion), as well as focal adhesion and actin cytoskeleton regulation. In sex contrasts, enriched categories remained centered on signaling and structural remodeling, with HM vs. HF showing stronger representation of axon guidance/synapse-related pathways and ECM-cell interaction, whereas LM vs. LF additionally involved Hippo signaling and immune-associated pathways (e.g., chemokine signaling and complement/coagulation). Overall, these enrichments suggest that temperature- and sex-associated CG methylation changes primarily target signaling networks and cytoskeletal/adhesion remodeling, with additional immune and endocrine regulation under chronic heat stress.

### 3.5. Joint Analysis of DNA Methylation and Transcriptomic Data

Integrative analysis cross-referenced DEGs with CG-DMGs to identify genes showing concurrent transcriptional and CG methylation changes (Table 3). The largest overlap (DEGs ∩ CG-DMGs) was detected in females under heat stress (LF vs. HF, 624 genes) (Table S3), followed by males (LM vs. HM, 177 genes) (Table S4) and the sex contrast under heat stress (HM vs. HF, 45 genes) (Table S5), whereas no overlapping genes were observed under low temperature (LM vs. LF). Most overlapping genes were linked to CG-DMRs located in gene bodies, indicating that heat- and sex-associated transcriptional variation is frequently accompanied by CG methylation differences, particularly within gene bodies.

The directionality between expression change (E) and methylation change (M) was further summarized by genomic region (Table 4). In both within-sex heat contrasts, the dominant pattern was downregulation coupled with hypermethylation (E− & M+), especially within gene bodies (LF vs. HF: 509 genes; LM vs. HM: 170 genes), with additional genes showing upregulation with hypermethylation (E+ & M+) and smaller proportions displaying methylation–expression discordance (E+ & M− or E− & M−). In contrast, the heat-stress sex comparison (HM vs. HF) involved fewer overlapping genes and showed only E+ categories (E+ & M+ and E+ & M−), mainly in gene bodies, suggesting a distinct coupling pattern for sex-biased transcription under heat. In summary, both sexes are dominated by downregulated genes coupled with hypermethylation, but this pattern is even more dominant in males, while females show more mixed patterns and broader remodeling.

**Figure 5:**
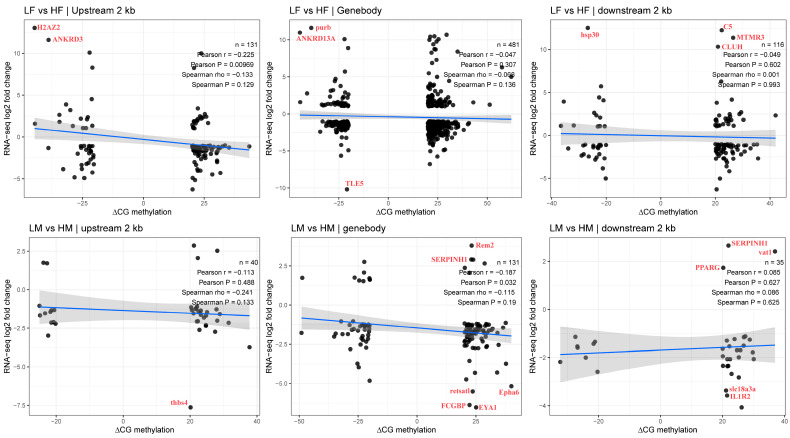
Correlation between regional CG methylation changes and gene expression changes of overlapping genes (DEGs ∩ CG-DMGs) in the LF vs. HF and LM vs. HM comparisons. Scatter plots were generated separately for the upstream 2 kb, gene body, and downstream 2 kb regions, with each point representing one overlapping gene. The *x*-axis indicates CG methylation difference (ΔCG methylation), and the *y*-axis indicates RNA-seq expression change [log2(fold change)]. Blue lines represent linear regression fits with shaded 95% confidence intervals. Pearson and Spearman correlation coefficients and corresponding *p* values are shown in each panel. Representative outlier genes with large concurrent methylation and expression changes are highlighted in red.

Correlation analyses between regional CG methylation changes and expression changes of overlap genes in the LF vs. HF and LM vs. HM comparisons revealed generally weak associations, with the clearest pattern observed in the female upstream 2 kb region (Figure 5). Nevertheless, several genes were visually distinguishable as prominent outliers in the scatter plots, exhibiting large concurrent changes in both CG methylation and gene expression (Table 5). These genes were located far from the origin and were mainly distributed in biologically interpretable quadrants representing upregulation with hypomethylation (E+ & M−) or downregulation with hypermethylation (E− & M+), particularly in the upstream 2 kb region. In the LF vs. HF comparison, two chrW-linked genes, H2AZ2 and ANKRD13A, displayed notable E+ & M− patterns in the upstream 2 kb region. In addition, ANKRD13A and purb in the gene body, as well as hsp30 in the downstream 2 kb region, also showed strong E+ & M− patterns. In the LM vs. HM comparison, thbs4 in the upstream 2 kb region exhibited a marked E− & M+ pattern, whereas Epha6, EYA1, and FCGBP in the gene body also showed clear E− & M+ relationships. By contrast, IL1R2 and slc18a3a in the downstream 2 kb region displayed notable E+ & M− patterns. These outlier genes may represent candidate genes associated with sex-related methylation–expression responses to chronic heat stress in tongue sole liver.

Functional enrichment of the overlapping genes highlighted pronounced sex-dependent epigenetic-transcriptional coupling. In females (LF vs. HF), the overlap set was strongly enriched for immune and inflammatory processes (e.g., innate immune response, leukocyte activation, cytokine regulation) together with lipid-related functions (e.g., response to fatty acid) (Table S6). Consistently, KEGG pathways were significantly enriched (q-value < 0.05) for host-defense and inflammatory signaling (NOD-like receptor, Toll-like receptor, RIG-I-like receptor, chemokine signaling, NF-κB and IL-17 signaling, Fc gamma R-mediated phagocytosis), as well as liver-relevant metabolic and detoxification pathways (drug metabolism-cytochrome P450, cholesterol metabolism, lipid and atherosclerosis, alcoholic liver disease) (Table S7), indicating coordinated immune–metabolic remodeling accompanied by CG methylation changes. In males (LM vs. HM), enriched GO terms were fewer and mainly related to immune cell maintenance/proliferation, with additional DNA recombination/replication-associated terms (Table S8); KEGG signals (e.g., insulin signaling, non-homologous end joining, proteasome, ferroptosis, steroid biosynthesis) were nominal but not significant after multiple-testing correction (q-value > 0.05) (Table S9), consistent with a weaker or more restricted coupled response.

### 3.6. Summary of Sex-Associated Overlap-Gene Patterns Under Chronic Heat Stress

A synthesis of overlap genes showing concurrent transcriptional and CG methylation changes (DEGs intersect CG-DMGs) suggests sex-associated candidate regulatory patterns in tongue sole liver under chronic heat stress (Table 6). Females (LF vs. HF) displayed a larger overlap set (624 genes; 1283 DMR-gene records) than males (LM vs. HM; 177 genes; 375 records), with more genes carrying CG-DMRs in multiple genomic regions, consistent with broader methylation-associated transcriptional remodeling in females. In both sexes, overlap genes were enriched for gene-body CG-DMR associations and were dominated by the E − &M+ pattern (downregulation coupled with CG hypermethylation), suggesting a recurring methylation–expression association under heat exposure. However, the functional composition differed: females showed enrichment for immune–inflammatory signaling together with liver metabolic/detoxification programs (e.g., innate/NF-kappaB-related regulators, lipid/cholesterol handling, and xenobiotic metabolism), whereas males were comparatively enriched for metabolic control, proteostasis/autophagy, and DNA replication/repair pathways, pointing to potentially distinct sex-dependent modes of hepatic thermal adaptation.

Notably, female-associated patterns also included coordinated changes in W-linked genes. A set of 24 chrW overlap genes (41 records) showed transcriptional upregulation in HF relative to LF, accompanied predominantly by gene-body CG hypermethylation (23/41 gene-body; 29/41 hypermethylated). These W-linked candidates include immune effectors (e.g., C5), chromatin/epigenetic regulators (H2AZ2, NSD3, SUDS3), and genes involved in cellular remodeling and stress responses (MTMR3, WDFY3, RASA1, LIMK2, KIF2A, TNKS, GIT2). These results suggest that chronic heat stress may involve a W-chromosome-associated molecular component contributing to the stronger female-biased response under elevated temperature, although functional validation is required.

## 4. Discussion

### 4.1. Sex-Specific Hepatic Responses to Chronic Heat Stress in Tongue Sole

The present study suggests that chronic heat stress is associated with different molecular responses in female and male tongue sole liver. Females exhibited broader sets of transcriptomic and methylomic changes than males, including larger sets of DEGs, CG-DMGs, and overlap genes, suggesting greater molecular plasticity under sustained thermal challenge. This pattern is biologically plausible in tongue sole, where females are the larger and faster-growing sex and where sex-related developmental regulation is tightly linked to temperature and DNA methylation [41]. More generally, temperature-sensitive changes in DNA methylation and gene expression have been documented in several teleost fishes, supporting the view that epigenetic remodeling is an important component of vertebrate responses to altered thermal environments [28,56,57,58]. However, because this study used a limited number of individuals per sex-temperature group, these sex-associated patterns should be regarded as candidate molecular signatures that require validation in larger, independently replicated populations.

A notable implication of this pattern is that sex should be treated as a major biological variable in studies of thermal resilience in tongue sole. If males and females recruit partly different pathways under heat conditions, pooling the sexes may obscure both the mechanistic basis of thermal response and informative candidate markers. This is particularly relevant for tongue sole because temperature-sensitive sex-related regulation has already been shown to involve methylation-dependent processes, and because female performance is of special economic value in aquaculture. Thus, the sex-associated patterns observed here provide a useful basis for designing future studies with larger sample sizes, replicated tanks, and sex-stratified validation.

### 4.2. CG Methylation as a Major Epigenetic Layer Associated with Heat-Responsive Gene Regulation

A second major finding of this study is that the heat-responsive methylation signal was dominated by the CG context, both at the DMC/DMR level and in downstream integrative analyses. This agrees with the broader vertebrate pattern in which most CpG sites are methylated genome-wide, whereas hypomethylated CpG-rich regions are concentrated in specific regulatory features such as CpG islands, promoters, and enhancers [59,60]. Promoter or promoter-proximal methylation is classically associated with transcriptional repression, whereas intragenic methylation often shows weaker, mixed, or context-dependent associations with gene expression [61,62]. Accordingly, the modest negative correlations observed in part of the present dataset are biologically plausible and are consistent with possible involvement of CG methylation in shaping heat-responsive transcription. At the same time, these correlations do not demonstrate direct causality and indicate that methylation is only one component of a broader regulatory framework that includes transcription factors, chromatin remodeling, endocrine signaling, and post-transcriptional control.

The predominance of gene-body CG-DMR associations among overlapping genes is also noteworthy. Although promoter methylation is often easier to interpret mechanistically, growing evidence from vertebrates and teleosts suggests that methylation downstream of the transcription start site, including first introns and other intragenic regions, can also be informative for gene regulation [44,62,63]. In this study, the enrichment of heat-responsive overlap genes in gene-body CG-DMRs suggests that chronic heat stress may be associated not only with promoter-proximal methylation changes but also with broader intragenic methylation patterns. This interpretation is consistent with studies in fish showing that methylation–expression relationships depend strongly on genomic context and that, even when genome-wide coupling is weak, biologically meaningful associations can still be detected for subsets of responsive genes [63,64,65].

### 4.3. Female-Biased Immune–Metabolic Remodeling Versus Male-Biased Homeostasis and Repair

The joint methylome–transcriptome analyses suggest that females and males deploy fundamentally different hepatic response modes under chronic heat stress. In females, overlap genes and pathway enrichment converged strongly on innate immune activation, inflammatory signaling, lipid/cholesterol metabolism, and xenobiotic or detoxification functions. This pattern points to a broad immune–metabolic remodeling response, in which inflammatory pathways and energy-handling pathways are activated together. Such coupling is biologically coherent because activation of immune responses is metabolically costly and closely integrated with lipid metabolism and inflammatory regulation, with PPARG representing one plausible transcriptional node linking these processes [66,67,68].

By contrast, the male response was more restricted and was enriched for proteostasis, autophagy, DNA replication/repair, and metabolic maintenance. Candidate genes such as SERPINH1/HSP47, ATG7, proteasome-related genes, and DNA repair factors fit well within this interpretation, as these functions are typically associated with protein quality control, extracellular matrix maintenance, and genome stability under cellular stress [69,70,71,72]. This suggests a more conservative strategy centered on preserving cellular homeostasis rather than mounting a broad inflammatory-metabolic reprogramming. From a mechanistic perspective, these male-biased pathways may reflect prioritization of survival and structural integrity, whereas the female-biased program may reflect a more dynamic but also more energetically costly adjustment to long-term heat exposure.

### 4.4. Potential Contribution of W-Linked Genes to Female-Specific Heat Responses

An especially interesting feature of the present study is the identification of female-specific overlap genes on the W chromosome, including candidates such as H2AZ2, ANKRD13A, purb, and C5. These genes may provide a potential link between sex chromosome biology and heat-associated epigenetic-transcriptional responses. In tongue sole, several female-specific W-linked genes have been implicated in sex-biased biological processes. For example, zbed1 copies specific to chromosome W have been shown to be essential for female-biased sexual size dimorphism, and ewsr1-w has been implicated in ovarian development through female-biased expression and functional analyses [73,74]. Moreover, DNA methylation has been demonstrated to play crucial roles in sex-related processes, including temperature-induced sex reversal, in tongue sole [41]. These observations support the hypothesis that chronic heat stress may involve a W chromosome-associated component, but the W-linked genes identified here should be regarded as candidates until functional validation is available.

Within this framework, the E+ & M− pattern of W-linked genes such as H2AZ2 is noteworthy. H2A.Z histone variants are broadly involved in chromatin-based regulation of transcription, replication, and DNA repair and have also been linked to environmentally responsive gene expression in other systems [75,76,77]. It is therefore plausible that activation of W-linked chromatin regulators under heat stress may contribute to the broader female-biased transcriptional plasticity observed in this study. Nevertheless, functional validation will be required before these genes can be considered confirmed regulators of female-biased heat responses.

### 4.5. Implications for Heat-Resilience Breeding in Tongue Sole

From a breeding perspective, these findings provide several entry points for improving heat resilience in tongue sole. First, the present results reinforce that thermal tolerance should be considered a complex, polygenic, and sex-dependent trait, rather than inferred from one or a few stress markers alone. Second, the overlap genes identified here, particularly those showing large coupled changes in CG methylation and expression, and especially those associated with promoter-proximal regions, represent promising candidates for biomarker discovery and downstream validation. Genes such as H2AZ2, ANKRD13A, PPARG, TNF, IKBKB, and SERPINH1 are of particular interest because they connect directly to the major biological axes uncovered in this study, including chromatin remodeling, immune signaling, lipid metabolism, detoxification, and proteostasis. More broadly, previous studies have shown that genomic selection is increasingly feasible in aquaculture species, including for complex resilience traits such as heat tolerance, and recent reviews further suggest that integrating multi-omics information can improve the prioritization of candidate loci and the identification of breeding targets [78,79,80].

At the same time, the present data argue for caution and should be viewed as a preliminary baseline for future validation. The methylation–expression correlations detected here were generally modest, and the study was limited by two biological replicates per sex-temperature group, one tank per temperature treatment, and the absence of direct measurements of growth performance, survival rate, and liver histology. Therefore, the candidate genes identified here should not be treated as deterministic markers without further testing in larger populations, replicated tank designs, and breeding-relevant conditions. A realistic next step would be to validate the highlighted overlap genes in expanded populations, assess whether their expression or methylation states are associated with survival, growth, physiological tolerance, and liver tissue integrity under heat stress, and determine whether sex-specific prediction models outperform pooled models. In the longer term, combining genome-wide markers with transcriptomic, epigenetic, and performance data may help develop breeding strategies that are both more precise and more robust to future climatic warming in tongue sole aquaculture. Given the sex bias observed here and the production preference for females in tongue sole aquaculture, incorporating validated sex-specific molecular signatures into future breeding pipelines may improve the selection of heat-resilient broodstock.

## 5. Conclusions

This exploratory study provides a preliminary baseline for understanding transcriptomic and methylomic responses to chronic heat stress in tongue sole liver and suggests that these responses are sex-associated. Females showed broader transcriptional and CG methylation-associated changes than males, with a larger set of overlap genes linking expression variation and CG methylation differences. These female-biased candidate responses were mainly associated with immune–inflammatory signaling, lipid metabolism, and detoxification, whereas males showed a more restricted response centered on proteostasis, autophagy, and genome maintenance. W-linked genes such as H2AZ2 and ANKRD13A emerged as promising candidates that may connect sex chromosome biology with heat-associated molecular responses, but functional validation and larger replicated studies are required before firm conclusions can be drawn. Overall, the results provide candidate genes and pathways for future studies of thermal adaptation and heat-resilient breeding in tongue sole.

## Figures and Tables

**Table 1:** Summary of global DNA methylation levels across cytosine contexts in liver samples of tongue sole. C indicates all cytosine contexts; CG indicates cytosine-guanine sites; CHG and CHH indicate non-CG contexts, where H represents A, C, or T. HM and HF represent males and females under high-temperature conditions, respectively, whereas LM and LF represent males and females under low-temperature conditions. Values are percentages (%).

Sample	C (%)	CG (%)	CHG (%)	CHH (%)
HF1	9.63	72.59	2.44	2.49
HF2	8.8	71.65	1.26	1.41
HM1	9.26	73.06	2.01	2.03
HM2	9.33	73.43	1.99	2.00
LF1	8.74	68.58	1.57	1.65
LF2	8.66	70.1	1.5	1.57
LM1	8.62	70.23	1.31	1.39
LM2	9.36	73.05	2.02	2.06

**Table 2:** Number of differentially methylated genes (DMGs) identified across four pairwise comparisons in different methylation contexts. C indicates all cytosine contexts; CG indicates cytosine-guanine sites; CHG and CHH indicate non-CG contexts, where H represents A, C, or T. DMGs were defined as genes associated with differentially methylated regions (DMRs) in the corresponding context. HM and HF represent males and females under high-temperature conditions, respectively, whereas LM and LF represent males and females under low-temperature conditions.

Group	C	CG	CHG	CHH
HM vs. HF	235	6272	53	1940
LF vs. HF	247	7356	57	1969
LM vs. HM	231	6824	52	1650
LM vs. LF	142	4703	38	1422

**Table 3:** Number of overlapping genes between differentially expressed genes (DEGs) and CG-associated differentially methylated genes (CG-DMGs) across four pairwise comparisons. Overlapping genes were classified according to the genomic location of the associated CG-DMRs, including downstream 2 kb, gene body, and upstream 2 kb regions. The All column represents the total number of unique overlapping genes in each comparison. Because a gene may contain CG-DMRs in more than one genomic region, the sum of regional counts does not necessarily equal the total number in All. HM and HF represent males and females under high-temperature conditions, respectively, whereas LM and LF represent males and females under low-temperature conditions.

Group	Downstream 2 kb	Gene Body	Upstream 2 kb	All
HM vs. HF	5	32	11	45
LF vs. HF	116	481	131	624
LM vs. HM	35	131	40	177
LM vs. LF	0	0	0	0

**Table 4:** Distribution of overlapping genes (DEGs intersect CG-DMGs) according to the direction of gene expression and CG methylation changes across genomic regions. E+ and E− indicate higher and lower gene expression, respectively, in the second group relative to the first group in each pairwise comparison; M+ and M− indicate higher and lower CG methylation, respectively, using the same direction. Thus, for LF vs. HF and LM vs. HM, positive values refer to the high-temperature group relative to the low-temperature group within each sex. Counts are shown for genes associated with CG-DMRs in the downstream 2 kb, gene body, and upstream 2 kb regions, as well as the total number of records in each comparison. HM and HF represent males and females under high-temperature conditions, respectively, whereas LM and LF represent males and females under low-temperature conditions.

Group	Trend	Downstream 2 kb	Gene Body	Upstream 2 kb	Total
HM vs. HF	E+ & M+	3	24	0	27
	E+ & M−	5	45	17	67
	E− & M+	0	0	0	0
	E− & M−	0	0	0	0
LF vs. HF	E+ & M+	42	161	37	240
	E+ & M−	22	82	28	132
	E− & M+	78	509	91	678
	E− & M−	29	156	48	233
LM vs. HM	E+ & M+	4	13	6	23
	E+ & M−	0	18	4	22
	E− & M+	30	170	34	234
	E− & M−	11	72	13	96

**Table 5:** Representative overlapping genes (DEGs intersect CG-DMGs) prioritized from the six regional methylation–expression correlation plots based on extreme concurrent changes in transcription and CG methylation. Genes were selected as visually prominent outliers showing large absolute values of log2(fc) and delta methylation and mainly exhibiting biologically interpretable patterns such as upregulation with hypomethylation (E+ & M−) or downregulation with hypermethylation (E− & M+). Region indicates the genomic location of the associated CG-DMR(s). log2(fc) represents the RNA-seq expression change and delta methylation represents the CG methylation difference, both calculated as the second group relative to the first group in each pairwise comparison; FDR and q-value indicate the adjusted significance of differential expression and differential methylation, respectively. HM and HF represent males and females under high-temperature conditions, respectively, whereas LM and LF represent males and females under low-temperature conditions.

Group	chr	Symbol	Region	log2(fc)	FDR	Δmethy	q-Value
LF vs. HF	W	H2AZ2	Upstream 2 kb	13.05	0.00	−44.65	0.00
	W	ANKRD13A	Upstream 2 kb, Gene body	11.61	0.00	−38.92	0.00
	W	purb	Gene body	10.97	0.00	−44.66	0.00
	9	TLE5	Gene body	−10.19	0.04	−20.87	0.02
	3	hsp30	Downstream 2 kb	12.54	0.00	−26.76	0.00
	W	C5	Downstream 2 kb	12.26	0.00	22.35	0.00
	W	MTMR3	Downstream 2 kb	11.38	0.00	26.53	0.00
	W	CLUH	Downstream 2 kb	10.34	0.00	20.96	0.00
LM vs. HM	9	thbs4	Upstream 2 kb	−7.63	0.01	20.14	0.01
	5	Epha6	Gene body	−5.18	0.00	40.04	0.00
	19	EYA1	Gene body	−6.52	0.01	25.08	0.01
	1	FCGBP	Gene body	−6.37	0.01	22.28	0.03
	2	retsatl	Gene body	−5.51	0.00	23.63	0.00
	18	Rem2	Gene body	3.79	0.04	23.17	0.00
	1	SERPINH1 (HSP47)	Gene body	2.91	0.01	23.03	0.02
	15	IL1R2	Downstream 2 kb	−3.58	0.00	21.50	0.02
	14	slc18a3a	Downstream 2 kb	−3.37	0.00	21.16	0.02
	11	SERPINH1	Downstream 2 kb	2.66	0.00	21.94	0.00
	2	vat1	Downstream 2 kb	2.41	0.00	36.90	0.00
	6	PPARG	Downstream 2 kb	1.73	0.02	20.27	0.00

**Table 6:** Sex-specific characteristics of heat-responsive overlap genes (DEGs ∩ CG-DMGs) in tongue sole liver. HM and HF represent males and females under high-temperature conditions, respectively, whereas LM and LF represent males and females under low-temperature conditions.

Feature	Female (LF vs. HF)	Male (LM vs. HM)	Interpretation
Unique overlap genes	624	177	Females show a substantially larger set of genes with concurrent expression and CG methylation changes under chronic heat stress.
CG-DMGs	1283	375	Indicates more CG-DMR associations per gene in females (more extensive remodeling).
CG-DMR location (unique genes)	Gene body: 481; Upstream 2 kb: 131; Downstream 2 kb: 116	Gene body: 131; Upstream 2 kb: 40; Downstream 2 kb: 35	In both sexes, overlap genes are enriched for gene-body CG-DMRs, consistent with gene-body methylation being the dominant coupling signal.
Genes with CG-DMRs in ≥2 regions	98 (≥3 regions: 6)	28 (≥3 regions: 1)	Females show more genes with multi-region CG methylation changes, suggesting broader regulatory remodeling.
Dominant E − M pattern (record-level)	E − &M+ = 52.8%	E − &M+ = 62.4%	Both sexes show a dominant trend of downregulation coupled with hypermethylation, more pronounced in males.
Representative functional themes/genes	Immune–inflammatory and liver metabolic/detox modules (e.g., TNF, IKBKB, NFKBIA, IRF5, TBK1, JAK/STAT, plus UGTs/FMO/GSTA, and lipid/cholesterol genes such as PPARG, ABCA1, LDLR, ANGPTL4, CYP7A1)	Metabolic control + proteostasis + genome maintenance (e.g., IRS2, PPARG, HKDC1, EIF4EBP1; proteasome/autophagy genes; DNA replication/repair genes such as XRCC5, PRKDC, MCMs, POLE)	Females show broader immune–metabolic and detox-associated remodeling; males show a comparatively focused overlap set enriched for cellular homeostasis modules.
Shared overlap genes (core)	-	-	51 shared genes, including PPARG and SOCS3, suggesting a small conserved epigenetic-transcriptional response across sexes.

## Data Availability

The raw RNA-seq and WGBS data generated from eight liver samples under low and high temperature conditions were deposited in the CNCB database (https://www.cncb.ac.cn/) with accession numbers CRA030694 and CRA032139, respectively.

## Supplementary Materials

The following supporting information can be downloaded at: https://www.mdpi.com/article/10.3390/ani16132078/s1, Table S1: Summary statistics of WGBS data generated from liver samples of tongue sole under low- and high-temperature conditions; Table S2: Summary statistics of RNA-seq data generated from liver samples of tongue sole under low- and high-temperature conditions; Table S3: Detailed information for overlapping genes between differentially expressed genes (DEGs) and CG-associated differentially methylated genes (CG-DMGs) in the LF vs. HF comparison; Table S4: Detailed information for overlapping genes between differentially expressed genes (DEGs) and CG-associated differentially methylated genes (CG-DMGs) in the LM vs. HM comparison; Table S5: Detailed information for overlapping genes between differentially expressed genes (DEGs) and CG-associated differentially methylated genes (CG-DMGs) in the HM vs. HF comparison; Table S6: Gene Ontology (GO) enrichment results for overlapping genes between DEGs and CG-DMGs in the LF vs. HF comparison; Table S7: Kyoto Encyclopedia of Genes and Genomes (KEGG) enrichment results for overlapping genes between DEGs and CG-DMGs in the LF vs. HF comparison; Table S8: Gene Ontology (GO) enrichment results for overlapping genes between DEGs and CG-DMGs in the LM vs. HM comparison; Table S9: Kyoto Encyclopedia of Genes and Genomes (KEGG) enrichment results for overlapping genes between DEGs and CG-DMGs in the LM vs. HM comparison.
